# Uncovering the lipidic basis for the preparation of functional nicotinic acetylcholine receptor detergent complexes for structural studies

**DOI:** 10.1038/srep32766

**Published:** 2016-09-19

**Authors:** Orestes Quesada, Carol González -Freire, María Carla Ferrer, José O. Colón -Sáez, Emily Fernández-García, Juan Mercado, Alejandro Dávila, Reginald Morales, José A. Lasalde-Dominicci

**Affiliations:** 1 Department of Physical Sciences, University of Puerto Rico, Río Piedras Campus, PO Box 23323, San Juan, 00931-03323, Puerto Rico; 2 Department of Chemistry, University of Puerto Rico, Río Piedras Campus, PO Box 23346, San Juan, 00931-3346, Puerto Rico; 3 Department of Biology, University of Puerto Rico, Río Piedras Campus, PO Box 70377, San Juan, 00936-8377, Puerto Rico; 4 Department of Pharmaceutical Sciences, School of Pharmacy, University of Puerto Rico, San Juan, 00936, Puerto Rico; 5 University of Puerto Rico, Molecular Sciences and Research Center, San Juan, 00926, Puerto Rico

## Abstract

This study compares the lipid composition, including individual phospholipid molecular species of solubilized nAChR detergent complexes (nAChR-DCs) with those of the bulk lipids from their source, *Torpedo californica* (*Tc*) electric tissue. This lipidomic analysis revealed seventy-seven (77) phospholipid species in the *Tc* tissue. Analysis of affinity-purified nAChR-DCs prepared with C-12 to C-16 phospholipid analog detergents alkylphosphocholine (FC) and lysofoscholine (LFC) demonstrated that nAChR-DCs prepared with FC12, LFC14, and LFC16 contained >60 phospholipids/nAChR, which was more than twice of those prepared with FC14, FC16, and LFC12. Significantly, all the nAChR-DCs lacked ethanolamine and anionic phospholipids, contained only four cholesterol molecules, and a limited number of phospholipid molecular species per nAChR. Upon incorporation into oocytes, FC12 produce significant functionality, whereas LFC14 and LFC16 nAChR-DCs displayed an increased functionality as compared to the crude *Tc* membrane. All three nAChR-DCs displayed different degrees of alterations in macroscopic activation and desensitization kinetics.

The manner by which detergents affect lipid composition, functionality and stability of solubilized membrane proteins is a poorly understood aspect of the structural biology of membrane proteins. The nicotinic acetylcholine receptor (nAChR) is a model transmembrane protein that has been widely used for the study of lipid-protein interactions. During several decades, independent research groups including those led by Mark McNamee, Francisco Barrantes, José González-Ros, Gregorio Fernández-Ballester, and John Baezinger have provided much of the known information about the structural and functional basis for the lipid regulation of reconstituted *Torpedo californica* (*Tc*) nAChR ion channel function. Overall, these groups made two fundamental discoveries: (1) the nAChR is very sensitive to the lipid environment^1^,^2^ and (2) several lipid mixtures can preserve both nAChR ligand binding and ion channel activity^3^,^4^,^5^,^6^,^7^,^8^. The studies above have provided much of the current groundwork in the preparation of functional and stable nAChR-detergent complexes (nAChR-DCs) using lipid-analog detergents.

The elucidation of the dynamic detergent–lipid–protein interactions of solubilized membrane proteins remains a largely unexplored research field. Thus, this has led to the concept that detergents can alter the native lipid composition of solubilized membrane proteins, and this could lead to irreversible damage to the lipid-spanning domains and eventually functional loss, denaturation, and aggregation. In this scenario, detergent solubilization could compete or exclude a critical lipid species present in the native cell membrane that is essential for protein function or stability and results in a conformational change in the protein’s hydrophobic domains that could lead to irreversible partial and/or full denaturation of the protein and ultimate loss of function. There is little doubt that the chemical nature (size, critical micellar concentration, and functional groups) of the detergent is crucial in producing the appropriate lipid environment in the solubilized protein/detergent mixed micellar complex. The patterns observed in a model membrane protein such as the nAChR could be used to develop correlations in other structure-related membrane proteins^9^,^10^,^11^,^12^.

We have previously found that detergent structure is crucial in the solubilization of nAChR for the formation of nAChR-DCs that are stable and/or can be constituted into functional states^13^,^14^. These studies demonstrated that foscholine (FC) detergent could be effective in meeting these criteria. We further explored the effects of the detergent structure with two similar groups of these phospholipid- analog detergents, (FC and lysoFC (LFC) groups) with slightly different headgroups and acyl chains of 12, 14, and 16 carbon atoms^15^. In the LFC family detergent’s, we found that LFC14 and LFC16 were able to sustain the highest levels of functionality, displaying macroscopic responses similar to the nAChR in crude membranes, but that the shorter acyl chain LFC12 DC displayed a reduction in the nAChR channel function. Distinctively, in the FC family, only the shorter FC12 was able to sustain partial channel function (lacking fast activation), and the longer acyl chain containing FC14 and FC16 showed a significant reduction in nAChR channel activity.

Due to the significant effects of detergent acyl chain length that we observed and the possibility that these might involve detergent-induced delipidation, we have performed a comprehensive lipid analysis, that involve identification of individual phospholipid molecular species of the nAChR in its native environment and compare these to six nAChR detergent complexes prepared with phospholipid- analog detergents (LFC12, 14, 16, and FC12, 14, 16). Furthermore, we also performed a more uniform functional assessment of these nAChR-DCs in *Xenopus* oocytes using the two-electrode voltage clamp (TEVC) technique. The objectives of the study are to (1) investigate whether the nAChR-DCs prepared with these detergents reveal different lipid compositions, (2) define which lipid species are present in those nAChR-DCs that display functional activity and stability, and (3) ultimately, use this information to define conditions to prepare high-quality nAChR crystals for structural studies.

The main impediment towards achieving a high-resolution structure of the nAChR is the preparation of milligram amounts of homogeneous, functional, and stable nAChR-DCs^16^. Furthermore, high-resolution structural studies on membrane proteins like the nAChR have been hindered by the lack of structural models that could predict how detergents influence membrane-protein stability and function. To develop a more mechanistic approach to understand the action of detergents on membrane proteins, we must first elucidate the native lipid species that are essential to preserve functionality and stability in the protein-detergent-protein solubilized complexes. Admittedly, to allow for complex functional correlations, these studies would have to be expanded to include many different types of membrane proteins with many different detergents. In the present study, for the first time, we performed a comprehensive lipid analysis of six nAChR-DCs prepared with lipid-analog detergents. The present results provide information on the lipidic basis for the functionality and stability of nAChR-DCs. Moreover, these findings will provide a lipidomic strategy to prepare nAChR-DCs suitable for structural studies.

## Results

### Efficiency of Folch and the Bligh & Dyer methods in the extraction of *Tc* electric organ lipids

**Figure 1:**
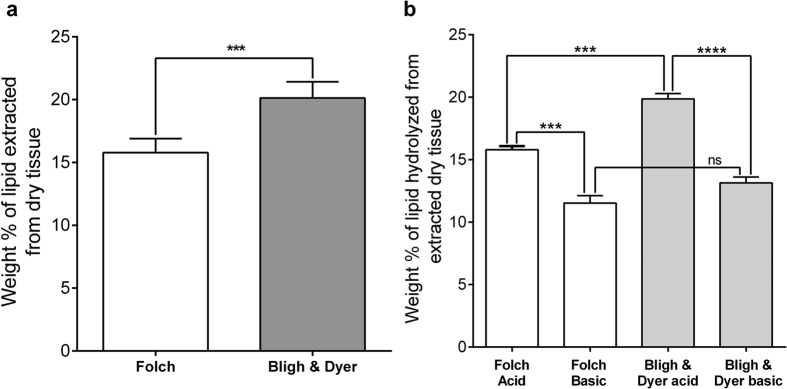
The effectiveness of Folch and D & B methods extracting lipids from *Tc* electric organ. Weight data represent the percent of lipid extracted from dry tissue. The percent of lipids from the *Tc* tissue was calculated using the values of mg of lipid/g dry mass of the tissue. (**a**) The percent of lipid recovery from the *Tc* electric organ were 15.79% and 20.14% for the Folch and B&D methods respectively. The data represent the mean ± SEM of six (6) independent extractions for each method compared using an unpaired t-test in Graph Pad Prism 6 (*P* < 0.0001). (**b**) The average weight percent of lipid extracted by the Folch and B&D after acidic hydrolysis were 15.79% and 19.86% respectively (*P* < 0.0001). The average weight percent of lipids for similar samples after base catalyzed hydrolysis were 11.52% and 13.13% respectively (*P* = 0.1182). The data represent the mean ± SEM of four (4) independent extractions for each experimental conditions using one-way ANOVA followed by Tukey’s multiple comparison test.

**Figure 2:**
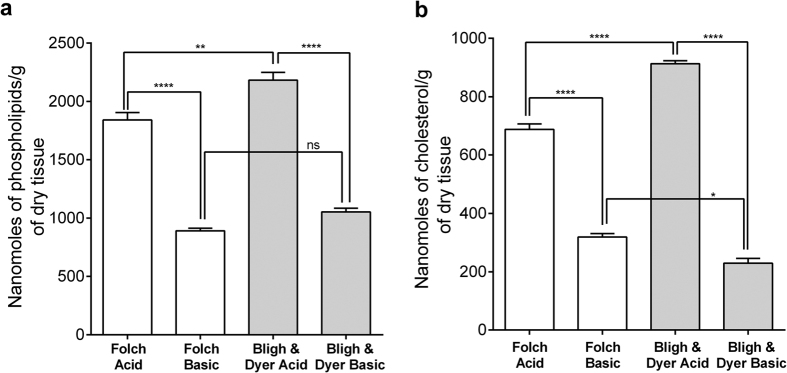
The effectiveness of the Folch and B&D methods extracting phospholipids and cholesterol from *Tc* electric organ. Comparison of phospholipid (**a**) and cholesterol (**b**) extraction from *Tc* electric organ using the Folch and B&D extraction methods under acidic and basic conditions. Total nanomoles of phospholipids and cholesterol recovered were expressed as mean ± SEM of four (4) independent extractions under acid or base catalyzed hydrolysis conditions and compared using one-way ANOVA followed by Tukey’s multiple comparison test.

Figure 1a shows the weight percent per dry tissue of total lipid extracted from *Tc* electric organ using two lipid extraction methods, Folch and Bligh & Dyer (B&D)^17^,^18^. Figure 1b shows the weight percent of total hydrolyzed lipid extracted from *Tc* electric organ using Folch and B&D, under acid and base-catalyzed hydrolysis conditions. Consistently, the B&D extraction was found to be more efficiently extracting cholesterol and phospholipids from *Tc* electric organ compared to the Folch method under the same experimental conditions (Fig. 2a,b). However, the difference between the two extraction methods was more significant under the acid-catalyzed hydrolysis. These results suggest that B&D was more efficient in the extraction of sphingolipids from *Tc* electric organ by almost 4%.

### Fatty acid profile of *Tc* electric organ lipids

The fatty acid profile of phospholipids extracted by Folch and B&D methods from *Tc* electric organs are displayed in Table 1, for acid and base-catalyzed hydrolysis. The total percent of saturated fatty acids were not significantly different (41.78% and 43.29%) between both methods under acid-catalyzed hydrolysis. The dominant saturated fatty acid for both Folch and B&D methods, under acid-catalyzed hydrolysis, were the palmitic (16:0) and stearic acids (18:0). However, the abundance of these saturated species under base-catalyzed hydrolysis range from 25% to 35% for the Folch and B&D extraction methods, respectively. On the other hand, the base-catalyzed hydrolysis for both extraction methods showed 16:0 as the only major saturated species. Furthermore, four different monoene species were also found in the *Tc* tissue extracted using both approaches, palmitoleic (16:1 *n*-7), oleic (18:1 *n*-9), erucic (22:1 *n*-9), and nervonic acid (24:1 *n*-9) acids; accounting for approximately 25% of the total fatty acids. The polyunsaturated fatty acids (PUFA) found in each extraction were; linoleic (18:2 *n*-3), eicosatrienoic (ETE, 20:3 *n*-3), eicosatetraenoic (ETA, 20:4 *n*-6), eicosapentaenoic (EPA, 20:5 *n*-3), docosapentaenoic (22:5 *n*-3), and docosahexaenoic (DHA, 22:6 *n*-3) acids, where DHA was the most abundant of this group. DHA represents 60 to 65% of the total PUFA as determined by both extraction methods. Thus, the *Tc* electric organ is rich in *n*-3 PUFA and has a very high *n*-3/*n*-6 ratio.

### Thin layer chromatography of total lipids extracted from *Tc* electric organ using Bligh and Dyer method

A qualitative assessment the of phospholipids composition in the lipid extract of the electric organ of *Tc* previous to a quantitative analysis was assayed using thin layer chromatography (TLC). The TLC lipid profile of freshly extracted *Tc* electric organ using the B&D method displayed eight (8) bands corresponding to the phospholipids PC, PE, PG, PI, PA, PS, SM, and cholesterol. Individual lipid components were identified by the comparison of their respective R_f_ values with those of the standards employed (see Supplementary Fig. 1).

### Analysis of phospholipid molecular species by UPLC ESI Q-Tof MS/MS

The molecular composition of all detected phospholipid classes from *Tc* electric organ or affinity-purified nAChR-DCs extracted using the B&D method were analyzed by UPLC ESI Q-Tof MS/MS (see Supplementary Fig. 2 and Supplementary Table 1). The major detected phospholipids in the ESI positive mode were PC, LPC, PE, LPE, and SM. From results, it is evident that the zwitterionic phosphatidylcholines with thirty-seven (37) molecular species are the most abundant, followed by the zwitterionic PE with twenty (20) different species and only five (5) SM species. Furthermore, the most abundant PC molecular species were PC 16:0/18:1 and PC O-16:0/16:1. On the other hand, the most abundant species of the PE’s group were PE 16:0/22:6 and the alkenyl/acyl PE 18:1/20:4. The phospholipid profile under ESI negative detection of the acid phospholipid fraction after Bond Elute separation process showed a discrete number of species being PG 16:0/22:6 the most abundant followed by PG 16:0/20:3 and PG 16:1/20:5 respectively. A total of eight (8) PG, seven (7) PI and four (4) PS species were detected under the experimental conditions used. See Supplementary Fig. 3 for a representative ESI-MS/MS spectrum for each type of phospholipids. The most abundant quasi-molecular ion [M-H]^−^ within the PI and PS groups were the PI 20:3/22:6 and PS 16:0/18:2. It is interesting to note that, except for PI, the most abundant phospholipids contain a more saturated sn-1 fatty acid.

The high susceptibility of the nAChR activity to the lipid environment has been well established^2^,^4^,^19^,^20^. It has long been recognized that the activity of nAChRs is affected upon detergent solubilization due to changes in the native lipid environment and/or delipidation of essential lipids. To obtain fully active and stable nAChR-DCs, we used a phospholipid-like detergent that resembles the most abundant endogenous native structure of those found in the *Tc* electric organ. Table 2 list the major detected phospholipids in the ESI positive mode for the nAChR-DCs of six phospholipid-like detergents with hydrophobic regions ranging from 12–16 carbon chain lengths. Most significantly, none of the DCs in the ESI negative mode indicated measurable levels of the anionic phospholipids found in the crude membrane preparation. In this study, it is assumed that the lipid content of the solubilized receptor/complex are the primary components being analyzed since extraction was carried out following affinity chromatography of the receptor complex.

**Figure 3:**
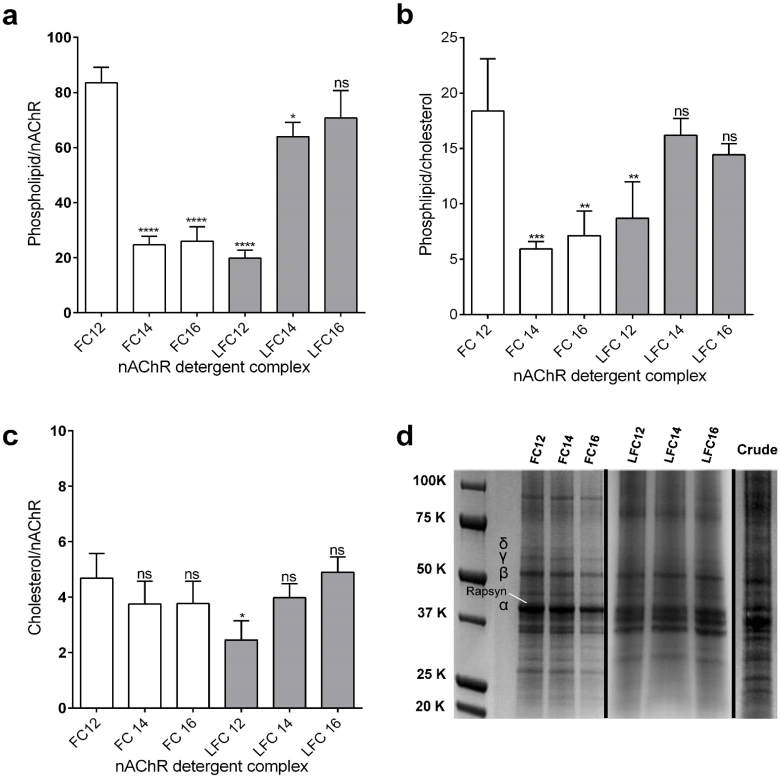
Effect of detergent in the amount of phospholipids, cholesterol and nAChR present in the detergent complex. Affinity purified nAChR detergent complex from the Foscholine (nAChR-FC12, LFC14 and nAChR-LFC16) families were analyzed as mentioned in methods section for; (**a**) phospholipid to nAChR ratio, **(b**) phospholipid to cholesterol ratio, (**c**) Cholesterol to nAChR ratio and (**d**) SDS-PAGE of *Tc* crude membranes and nAChR detergent complex for the FC and LFC family. The data represent the mean ± SEM of four (4) independent extractions for each experimental conditions using one-way ANOVA followed by Tukey’s multiple comparison test. Comparison to FC12 in (**a**) ****P < 0.0001, *P < 0.05. In (**b**) ***P < 0.0003, **P < 0.01. In (**c**) *P < 0.0182.

The phospholipids content was determined for each nAChR-DC (Fig. 3a), and as shown, detergents FC14, FC16, and LFC12 appear to remove associated phospholipids during the solubilization process. The FC12, LFC14, and LFC16 detergents were able to solubilize the nAChR from its scaffolding in the native *Tc* membrane with average phospholipids per nAChR of 83, 64, and 71 respectively. On the other hand, as expected, considering their higher phospholipid content, the same three detergents displayed a relatively high phospholipid: cholesterol ratio compared to the others and had an average value of 16 molecules of lipid per cholesterol (Fig. 3b). However, the cholesterol:nAChR ratio for all detergent complexes was found to have an average value of 3.9 molecules of cholesterol per nAChR (Fig. 3c), except for the FC12 complex that had ≈2.5 cholesterols per nAChR. The purity of *Tc* crude membranes and nAChR detergent complexes for the FC and LFC family was accessed qualitatively by SDS-PAGE. The gels indicate the presence of other proteins, primarily rapsyn (43-kD), but also a much lower level of ATPase (100-kD) protein, with the LFC family having lower levels of these contaminants (Fig. 3d).

### Two-electrode voltage clamp experiments (TEVC) in oocytes expressing nAChR-DCs

We took advantage of *Xenopus laevis* oocytes expression system, which has been previously shown to be able to incorporate pre-assembled nAChRs into their plasma membrane either in natural membrane vesicles or in reconstituted lipid vesicles, retaining nAChR’s native properties^21^. A 5-second application of ACh (100 μM) to an oocyte injected with crude membranes resulted in a mean amplitude of −275 ± 24 nA response (Table 3), whereas a 5-second pre-application of alpha-bungarotoxin (α-BTX, 1 μM) abolished this response completely, indicating that the ACh-evoked response was due to functional *Tc* nAChRs present in crude membrane preparations. Injection of oocytes with *Tc* nAChRs-DCs solubilized with members of the FC family, differing in the length of their acyl chain (C12-C16), resulted in responses with amplitudes that inversely depend on acyl chain length. FC12 displayed the highest mean amplitudes in this family (−217 ± 22 nA) when compared to the longer side chain, FC14 (−77 ± 18 nA), and FC16 (−28 ± 7 nA) (Table 3). Consistent with this finding, *Tc* nAChRs-DCs including members of the LFC family also showed a dependency on the length of the acyl chain. However, the effects the of the LFC’s were the opposite, with the shorter acyl chain LFC12 displaying the smallest mean amplitude (−25 ± 8 nA) as compared to LFC-14 (−420 ± 45 nA) and LFC-16 (−344 ± 25 nA) (Table 3).

## Discussion

A major obstacle towards attaining a high-resolution structure of the nAChR is the preparation of milligram amounts of functional, homogeneous, and stable nAChR-DCs. A critical aspect of the preparation of suitable nAChR-DCs for structural studies is the preservation of a lipid environment that maintains interactions with the protein that is essential to the functionality, stability, and ion channel machinery. Several efforts have been directed toward understanding the nature of these interactions, yet the specific elements responsible for maintaining functional activity of nAChR in the solubilized detergent-complex remain to be elucidated. Inherent to this understanding, is the possible problem of detergent delipidation or the exclusion of essential lipids from the nAChR-DCs. Herein, we describe substantial differences in lipid composition between affinity-purified detergent receptor complexes and that of receptor enriched crude membrane. We also discuss the differences in detergent-complex compositions that might result in conformational instability that could lead to non-functional receptors.

A pioneering study on the lipid composition of the electric organ in ray species, *Torpedo marmorata (Tm)*, demonstrated that the most abundant lipids were cholesterol, PC, PE, and SM^22^ and a later study identifies the acyl compositions of the different phospholipids in three ray species, including *Tc*, as well as nAChR enriched *Tm* membrane fragments^23^. The latter study also showed that tissue and membrane compositions were very similar. Since our study compared molecular species in tissue and solubilized nAChRs, we verify that the extraction approach was non-selective for the individual molecular species. To this end, four (4) independent extractions were carried out with both the Folch and B&D procedures using identical amounts of the *Tc* electric organ tissue. In general, these independent extractions produced consistent results for each method (Fig. 1a), but the total amount of lipid extracted was higher in the B&D extraction. The percent of lipids from the *Tc* electric tissue was calculated using the values of milligrams of lipid/g dry mass of the tissue. For the evaluation of these extraction methods, in term of lipid extraction efficiency toward sphingomyelin species, the extracted lipid samples were subjected to acid and basic-catalyzed hydrolysis conditions. The total percent of fatty acids recovered by each extraction method, (Fig. 1b), indicated that B&D method was more efficient for sphingolipids since the percent of amide containing species for lipids extracted with Folch and B&D methods were 4.27% and 6.72% respectively. A more effective phospholipid extraction could also explain the different phospholipid to cholesterol ratios of 2.67 and 2.40 found for the Folch and B&D extraction methods, respectively. Consistent with a previous study^23^, our results did not reveal differences in the identities of fatty acid methyl esters from *Tc* tissue (Table 1). However, the quantitative analysis of the fatty acids indicated that the levels of 22:6ω3 were much higher than those previously reported. The latter could be explained by seasonal differences in ray samples, but could also be due to the presence of antioxidants added in our extraction to protect this labile acid. In addition, even though the fatty aldehyde to acid ratio in PE was similar to that found in the previous study, MS analysis also showed that a substantial fraction (1/6) of the PC’s were certainly glycerol ethers (Supplementary Table 1).

In the nAChR-DCs lipid analysis, we found that those functionally stable (FC12, LFC14, and LFC16 DC’s), all contained sixty or more of the membrane phospholipid molecules per detergent complex, whereas those of the less functionally active (FC14, FC16, and LFC12 DC’s) contained less than half this number (Fig. 3a). The latter indicates that the higher level of phospholipid is essential for nAChR-DC stability or functionality. Also, except for LFC12 DC, which had an average of 2.46 cholesterol molecules per nAChR, all the other nAChR-DC’s had an average close to four (less than one per subunit), regardless of their subsequent functional reconstitution (Fig. 3c). This average value represents a significant (~74%) lower level of cholesterol compared to the previously estimated values from reconstituted *Tc* nAChR, clearly showing that a high level of cholesterol in the detergent solubilized nAChR was not required for maintaining a conformation that can be active when reconstituted into the oocyte plasma membrane^24^. In this respect, this is consistent with previous studies that demonstrated that several lipids and lipid mixtures could support allosteric transitions even in the absence of cholesterol^25^. Furthermore, since similarly solubilized nAChR’s were found to be stable in a lipid cubic phase for several weeks^15^, this also demonstrates that these nAChR-DC’s are very stable at much lower levels of cholesterol than previously considered necessary^24^.

Remarkably, the compositional analysis of all detergent complexes in this study indicated a somewhat surprising absence of detectable levels of the non-choline phospholipids that were present in the *Tc* tissue (see Supplementary Fig. 4). There is no simple explanation for the lack of PE or negatively-charged lipids in the nAChR-DCs, but it could be conjectured that these are not essential for the formation of stable mixed micelles containing appropriate levels of protein, lipid, and detergent during solubilization. In this regard, due to its smaller headgroup PE might not allow for appropriate micellar curvature and those with negatively charged headgroups could also be overwhelmed by the larger fraction of cationic species in *Tc* tissue. Nevertheless, their absence would appear to indicate that they are not necessary for the presence of a nAChR protein in the respective DC that is capable of specifically binding to carbamylcholine in affinity chromatography and to α-bungarotoxin.

Overall, the lipid composition found in the nAChR-DCs demonstrated a significant reduction in the number of lipid species compared to the bulk lipid composition in *Tc* membranes that also shows a degree of selectivity, since one would expect the complexes to have acyl compositions similar to that of the crude membrane preparation. Table 2 lists all of the lipid species detected in the six nAChR-DCs. The nAChR-LFC16 with 12 lipid species had the highest; LFC12 and LFC14, 11 lipid species; FC12 and FC16, 7 lipid species and FC14, 6 lipid species. Although the data shown in Table 2 is mainly qualitative, it is clear that the two nAChR-DCs (those of LFC16 and LFC14) that produced the highest functional ion channel activities among these nAChR-DCs contain the largest number of lipid species. There are two unique lipid species in the nAChR-LFC16 complex, PC14:0/18:2 and PC16:0/22:5, however, these are not present in two other functional detergent complexes (nAChR-LFC14 and nAChR-FC12). Likewise, since there are not any species detected in all of the most functional DC’s that are absent from any of the less functional DC’s, one can rule out the possibility that there are any specific species that are essential for the functionality when the DC’s are incorporated into oocytes.

On the other hand, a cross-correlation of the species found in the six DC’s with their abundance in the mass spectral analysis of *Tc* tissue phospholipids can be used to develop a better understanding of the possible discriminatory processes involved in the formation of the DC complexes. In this respect, one can either consider those species that are present in all of the complexes or those that are absent. All six DC’s contain the major tissue PC species (PC16:0/18:1, PC16:0/22:6 and PC(O-16:0:/16:1) and although this could be explained by their availability in the solubilization mixture, the same would not hold for PC(O-16:1/18:0) and PC(O-18:0/16:1) that are also present in all six DC’s, but are a minor tissue PC species. The possibility that glycerol ether PC lipid species are more tightly bound by the nAChR than diacyl-PC lipids would require further studies. The cross-correlation can also be used to determine those major *Tc* tissue membrane lipids that are absent from the six DC’s. Here, it is important to mention that in addition to the absence of detectable levels of non-choline phospholipids, the major sphingomyelin species, SM d18:1/16:0, is also missing from all six DC’s. There is no explanation for this observation, but its absence, as well as the presence in FC12, LFC14, and LFC16 DC’s of species that are found in trace amounts (PC 18:0/20:4, PC 20:4/20:5 and PC 20:5/20:4) in *Tc* tissue, would appear to show that the formation of nAChR/lipid/detergent complexes does some involve discriminatory processes with respect to lipid composition in these DC’s. These processes are not easily understood regarding our current knowledge of protein/lipid/detergent ternary complexes. However, one could propose a simple explanation for the lipid composition of the DC’s that involves the steric requirements for the formation of stable mixed-micellar complexes and the structural conformity of given species to these requirements. Still, further studies are necessary on the physical nature of these complexes.

In considering the use of oocyte injection to measure nAChR DC’s functionality, it was understood that there could be multiple routes or interactions that could affect the number of nAChR being expressed on the cell surface. Our results ruled out that chain length of the detergent could influence the incorporation of the nAChR-DC in the oocyte plasma membrane. Our first argument is that injection of nAChR crude (without detergent) gave a robust macroscopic current response in the *Xenopus* oocyte. This suggests that there is no need of detergent for the incorporation of the nAChR in the oocyte membrane. Moreover, as shown in Table 3, the normalized macroscopic current response of two detergent complexes with the same length (i.e. nAChR-FC12 and nAChR-LFC12) gave very different normalized macroscopic current responses. One could argue that if incorporation is influenced by the chain length of the detergent, these two nAChR-DCs would display similar macroscopic responses. The same pattern was observed for the nAChR-FC16 and nAChR-LFC16 detergents complexes which gave very different normalized macroscopic current responses of 0.08 and 1.25 respectively. In addition, we examined the phospholipid analog detergent 1-palmitoyl-2-hydroxy-sn-glycero-3-phospho-(10-rac-glycerol) (LFG-16), which produce a normalized macroscopic current response of 0.5^26^. Also, we demonstrated that nAChR-Cholate injected into *Xenopus* oocyte membrane produced a response similar to that of the nAChR present in the crude membranes^27^. Cholate has a much higher linear hydrophobic region than either of the phospholipid analog detergent families studied in this work. Overall, these results suggest that chain length of the detergent does not necessary affect the incorporation of the nAChR-DC in the oocyte membrane. Therefore, it was reasoned that given the structural similarities of the detergents tested, as well as the observed similarities in the lipid composition of the different complexes, there would be a limit on the number of different interactions that these DC’s might have with the oocyte’s cytoplasmic membranes and subsequently functionally expressed on the cell surface. In such a comparative study, the incorporation of the functional nAChR on the oocyte surface should be a good indicator of the functional integrity of the receptor in a given detergent complex. Indeed, injection of oocytes with *Tc* nAChRs-DCs solubilized using members of the lysofoscholine (LFC) family differing in their acyl chain lengths (C12-C16); resulted in responses in which the amplitudes depend on the length of the acyl chain: LFC12 displaying the smallest mean amplitudes when compared to the longer side chain, LFC14 and LFC16 (Table 3). To appropriately compare the variable amplitude of the different detergent complexes, we normalized them to the amplitude of the crude membranes. The results show that the longer acyl chain containing detergents in this family (LFC14 and LFC16) can support channel function better than the shorter side chain LFC12.

In addition, the activation kinetics are only significantly affected by the presence of the FC-12 detergent, although the increase in this parameter are present for all detergent complexes, none of the other detergents reach significance. Interestingly there were significant changes in the deactivation kinetics, with FC14 (3.3 ± 0.20 s) displaying faster deactivation than crude membranes (6.3 ± 0.50 s). Although both LFC and LFC16 form nAChR-DCs retain functionality to levels close to the nAChR in their native membranes, the differences in the properties of the responses, although none significant require further studies. These results illustrate the importance of detergent structure in the formation of nAChR-DCs and the need to assess receptor functionality following detergent extraction.

The lipid-protein interactions of the nAChR have been extensively studied using reconstituted membranes^6^,^7^,^20^,^28^,^29^,^30^,^31^. Titration of nAChR reconstituted into membranes composed of phosphatidylcholine/cholesterol by decreasing the lipid to protein ratio demonstrated that the nAChR functionality decreased rapidly under 65 lipids per receptor^24^. These values are in agreement with our findings (Fig. 3a,b and Table 3). The protein-detergent complex formed by LFC12 and FC14 and FC16 displayed a significant reduction in the phospholipid to nAChR ratio (Fig. 3). The *Tc* nAChR membrane contains a 35 mole % in cholesterol^32^. The cholesterol requirement for a functionally reconstituted nAChR using ESR spectroscopy indicated a value 15 cholesterol molecules per nAChR, which would suggest three cholesterol molecules per nAChR subunit^32^. Previous studies in which the protein-lipid interaction of nAChR from *Tm* electric organ was measured using spin-labeled phospholipids using electron spin resonance (ESR) spectroscopy reported a value of 38–46 lipids/nAChR for boundary lipids^32^,^33^. These lower values probably arise from a requirement of higher lipid numbers for mixed micelle stability than that required for annular interactions in bilayers. These values have been used to define the lipid compositions during the past three decades of studies on the functional consequences of the interaction of nAChR with the lipid environment^30^,^34^.

The hypothesis that membrane physical properties influence nAChR function has also been proposed^4^,^5^,^35^,^36^. These studies propose that membrane fluidity modulates the population of nAChRs in the resting and desensitized states, and also that anionic phospholipids are essential to stabilizing the functional response of the nAChR. Early reconstitution studies using *Tc* nAChR revealed the importance of zwitterionic phospholipids, cholesterol, and anionic phospholipids in the stabilization of the receptor in an activable resting conformation^6^,^24^,^37^,^38^,^39^, whereas in their absence, the *Tc* nAChR stabilizes in an “uncoupled” conformation, meaning that agonist binding does not result in channel gating^40^. Furthermore, it was proposed that without this stabilization, the solubilized *Tc* nAChR cannot undergo agonist-induced conformational changes^40^. In addition, a study by daCosta *et al*.^4^, further evaluated the hypothesis that physical properties of the lipid modulate nAChR conformational transitions^4^. This study showed that incorporation of nAChRs into phosphatidic acid-containing membranes leads to dramatic increases in both, lateral packing densities and the gel to liquid crystal phase transition temperatures of the reconstituted membranes. These results inspired the hypothesis that a particular coupling between nAChR and the phospholipid headgroup could be relevant to the modulation of nAChR function.

Cholesterol and other sterols have also been reported to affect the biophysical properties of the nAChR including allosteric binding transitions and ion channel function. Although initially, it was suggested that cholesterol was a requirement for allosteric transitions^5^,^25^, subsequent work demonstrated that several lipids and lipid mixtures could facilitate allosteric transitions even in the absence of cholesterol^25^. The major effect of cholesterol described in the reconstituted nAChR was on ion-channel function. For instance, an increase of agonist-induced cation flux by cholesterol was demonstrated in reconstituted vesicles depending on the type and composition of other lipids present^5^,^25^. These studies demonstrated marked cholesterol-dependent changes on ion gating of reconstituted nAChR-rich vesicles, however, the explanations on the magnitude and nature of this effect have not been defined yet.

The stability of nAChR-DC is a critical parameter for structural studies. Recently we found that the nAChR-LFC-16 is most the stable complex among the six examined in the present study using the lipidic cubic phase fluorescence recovery after photobleaching (LCP-FRAP) methodology^15^. In that study, the least stable complex in the FRAP experiment was the LFC12 DC, which is consistent with our finding that this detergent complex is one with the highest lipid depletion and it correlation with the stability of these complexes previously estimated in the FRAP experiment^15^.

Another fundamental aspect of the preparation of nAChR-DCs suitable for crystallization studies is the purity of the sample. One of the main contaminants present in the crude *Tc* membranes is the peripheral nAChR-associated proteins, mainly the 43-kDa rapsyn protein and ATPase. During the last three decades, several attempts have been made to crystallize the nAChR^41^,^42^. Hucho’s research group reported small crystals with negative control experiments but no X-ray diffractions while Stroud’s research group did obtain several X-ray diffraction patterns^41^. Nonetheless, there have been no reports involving nAChR-crystallization experiments for more than twenty years. A significant difference between the purification protocols used by Huchos’s and Stroud’s research groups was the method used to remove the peripheral nAChR-associated proteins, mainly the 43-kDa rapsyn protein and ATPase^42^. Hucho’s protocol used the chaotropic salt lithium diiodosalicylate and chromatography on a Cibacrone Blue column to remove the peripheral membrane proteins while Stroud’s protocol relied on the alkaline treatment of the partially purified nAChR sample. We have reproduced both protocols in our laboratory and multiple crystallization trials have been performed using both types of purified nAChR samples. Although Stroud’s protocol did produce some crystals, these were not of sufficient quality for structural studies. The present data shows that all the nAChR-LFC-DC’s are almost free of ATPase; however, rapsyn (43K) was present in all nAChR-DCs (Fig. 3d and Supplementary Fig. 5). Also, we have previously hypothesized that the presence of rapsyn (43K) could provide stability to the oligomer given that it holds interactions between intracellular domains, especially the large domain between the transmembrane M3 and M4^43^. The most suitable detergent to prepare high-quality nAChR-DCs for structural studies must preserve a functional conformation that can be locked with the use of specific antagonists, sustain a lipid environment that confers stability to the oligomer during the crystallization process and be highly pure. As shown in the present study, not all these three properties can be achieved by a single detergent, nonetheless, some of them can maintain at least two of these properties. Along these lines, nAChR-LFC16 is fully functional and the most stable complex among all six complexes but still contains some ATPase. “Based on the present results, we propose to enhance the purity of the nAChR-LFC16 complex via removal of peripheral proteins. We also propose to improve the functionality of those nAChR complexes, (FC14, FC16, and LCF12) by lipid supplementation to assure a high phospholipid/nAChR ratio. We have produced crystals for both nAChR-LFC16 and nAChR-FC12 using LCP lipid matrixes in our laboratory, and both complexes have consistently produced lamellar and fiber X-ray diffractions. In summary, these results uncover a mechanistic implication for the detergent–dependent lipid exclusion behind the preparation of highly functional nAChR-DCs suitable for structural studies.

In summary, the present study has performed, for the first time, a comparison of lipid species present in the *Tc* electric organ and six nAChR-DCs prepared with phospholipid-analog detergents. We have found that although some of these detergent complexes do not have the same composition as either that of the crude membrane or that expected from previous studies, they are fully functional when incorporated into membranes and highly stable in LCP. These studies should provide grounds for further studies of the nAChR-lipid interaction and a guide for the preparation of high-quality nAChR-DCs for structural studies. The preparation of new functional and stable membrane protein-DCs from natural and recombinant sources will also provide the groundwork to validate forthcoming developing technologies for the crystallization of large membrane protein complexes.

## Materials and Methods

### Reagents

All reagents were purchased from Sigma-Aldrich unless otherwise specified.

### Crude membrane isolation, detergent solubilization, and receptor purification

*Torpedo californica* electric organ tissue was obtained from Aquatic Research Consultants, San Pedro CA, USA. *Tc* crude membrane isolation, detergent solubilization, and receptor purification were carried out as described before^13^. This study analyzed the lipid composition of crude membrane and nAChR-DC for the following: n-dodecylphosphocholine (FC12), n-tetradecylphosphocholine (FC14), n-hexadecylphosphocholine (FC16), 1-dodecanoyl-*sn*-glycero-3-phosphocholine (LFC12) 1-tetradecanoyl-*sn*-glycero-3-phosphocholine (LFC14), 1-palmitoyl-2-hydroxy-sn-glycero-3-phosphocholine (LFC16). All detergents were obtained from Anatrace (Maumee, OH, USA).

### Isolation and analysis of phospholipids fatty acid components from *Tc* membranes or nAChR-detergent complex

All *Tc* electric organ tissues were obtained from different animals caught within a period of two months to avoid possible seasonal changes in lipid content. *Tc* whole tissue or affinity-purified nAChR-DC samples were subjected to lipid extractions using Folch or B&D methods using butylated hydroxytoluene (BHT; 2.9 × 10^−5^ M), followed by 3.5 h of reflux with MeOH/HCl or MeOH/1N KOH for phospholipid hydrolysis^17^,^18^. For efficiency comparison of both lipid extraction methods for whole intact tissue, the frozen electric organ from *Tc* was homogenized in a blender, lyophilized and weighed before being subjected to the corresponding extraction method. Lipid extracts were dried under nitrogen and dissolved in 10% MeOH/diethyl ether (DE) for methylation using 0.5 ml of diazomethane. After 20 min, the reaction mixture was extracted with petroleum ether (PEt) and assayed in rhodamine 6-G–stained silica gel thin-layer chromatography plates with PEt: DE (98:2 v/v) as the solvent, excising cholesterol and fatty acid methyl ester (FAME) bands for further analysis. Isolated FAMEs were analyzed with Agilent 6890N Gas Chromatograph with 5975 Mass Selective Detector, equipped with an Agilent DB-23 capillary column [(50%-cyanopropyl)-methylpolysiloxane capillary, 250 μm I.D., 0.15 μm film thickness; Agilent Santa Clara, CA, USA]. Samples were dissolved in n-hexane and injected manually (injector temperature 250 °C, oven temperature 130 °C), and after 5 min, a 3 °C per min. ramp was applied to 200 °C (1-min hold), resuming the ramp to 220 °C with a 10-min hold as the final step. FAME quantitation was performed with an internal standard of known concentration absent from the lipids in the *Tc* tissue (20:1Δ^11^), dividing the total FAME by two (2) and using appropriate control samples to eliminate free fatty acids and trace amounts of contaminants present in all solvents tested. Total phospholipid and cholesterol amounts for both crude membranes and affinity purified nAChR-DCs were normalized using protein concentration values in mg/ml obtained from BCA assay measurements.

### Thin layer chromatography of lipids

Phospholipids were resolved using commercially available thin layer chromatography (TLC) plates (20 × 20 cm) from Whatman, Fisher Scientific, MA, USA. Plates were coated with Partisil K6 60A high-purity silica gel with polarity for normal phase separation. The plates were preconditioned by running in chloroform: methanol (2:1) until the solution reached the top and then activated by keeping them in an oven (110 °C) for 30 min. Aliquots (10 μl) of standard solutions containing phospholipid, cholesterol, and a mixture of lipids extracted from *Tc* electric organ were deposited onto the plate and was allowed to dry for 3 min before developing. The plate was developed in chloroform: methanol: ammonium hydroxide (60:35:5) acid and run to the top. The plate was then placed in a chamber with iodine vapor for visualization. R_f_ values were calculated using the formula; R_f_ = distance traveled by the analyte/distance traveled by the mobile phase.

### Solid phase extraction of lipid samples (pre-cleaning)

Dry lipid extracts from *Tc* electric organ tissues using B&D method was dissolved in CHCl_3_ and further separated into different lipids species using solid-phase aminopropyl extraction cartridges (Bond Elut NH_2_, 100 mg-1 ml) following manufacturer’s indications. Briefly, after conditioning the column by passing 6 ml of hexane, 200 μl CHCl_3_ lipid extract was loaded followed by a sequential elution with four different eluents: 2 ml of CHCl_3_, 3 ml of diethyl ether with 2% acetic acid, 3 ml MeOH, and a final 3 ml of 0.05 M ammonium acetate in chloroform/methanol plus 2% (v/v) 28% aqueous ammonium solution. The four fractions collected contained the non-polar lipids and cholesterol, non-esterified fatty acids, non-acids phospholipids and acidic phospholipids respectively.

### *Tc* membrane cholesterol quantitation

The cholesterol isolated from the rhodamine 6G stained silica gel G plates was assayed using the Wako cholesterol E-Kit (Wako Chemicals, Richmond, VA, USA) according to the manufacturer’s indications.

### Analysis of phospholipid molecular species by Ultra Performance Liquid Chromatography (UPLC) coupled to electrospray ionization mass spectrometry (ESI-MS/MS)

Phospholipids were isolated from other lipids using elute Bond Elut NH_2_ as mentioned above. The cartridge columns were equilibrated with 3 ml of hexane. Samples of 3 μg were dissolved in 100 μl of chloroform. Then, the columns were rinsed with 3 ml of chloroform/2-propanol (2:1), followed by 3 ml of 2% acetic acid in diethyl ether and finally 3 ml of methanol. The methanol fractions (MFs) contained the phospholipids and were dried under a nitrogen atmosphere. The MFs were analyzed using UPLC-ESI-MSe or MS/MS with Acquity Ultra Performance Liquid Chromatographer (Acquity UPLC) coupling with an XEVO G2S quadrupole-time-of-flight mass spectrometry (QToF) from Waters Corp. The liquid chromatography was performed using an Acquity UPLC BEH HILIC (1.7 μm, 2.1 mm × 100 mm) column. The mobile phase A was 10 mM ammonium acetate in water at pH 3, adjusted using formic acid and mobile phase B was acetonitrile. The gradient was as follows: 0–0.1 min, 100% B; 0.1–0.5 min, 92% B; 0.5–15 min, 80% B and then back to 100% B at 15.1 min to re-equilibrate the column for about 1 min. The injection volume was 0.5 μl, and the flow rate was 0.3 μl/min^44^. ESI analysis was performed in positive resolution mode using the MSe continuum method. The instrument was calibrated with a sodium iodide standard solution (2 μg/μl) in 2-propanol/water (50:50). The voltages used were: capillary 3 kV, sampling cone 75 kV, and source offset 40 kV. The source temperature was 100 °C, and the desolvation temperature was 350 °C. The gasses flows were: cone 50 L/h and desolvation 800 L/h. The acquisition time was 15 min, mass range 50 Da to 1,100 Da and the collision energy ramp was 20V to 30V. Leucine enkephalin (2 ng/μl) was used as a reference, a capillary voltage of 2 kV and a flow rate of 3.0 μl/min were employed.

### Two-electrode voltage clamp (TEVC) of nAChR-detergent complexes

Oocytes were retrieved surgically from a mature *Xenopus laevis* frog and placed immediately in calcium-free OR-2 solution (82 mM NaCl, 2.5 mM KCl, 1 mM MgCl_2_, 5 mM HEPES; and adjusted to a pH of 7.6 with NaOH). Frogs were obtained from Xenopus Express Inc. FL, USA. All animal procedures were done in accordance with the guidelines and with the approval of the University of Puerto Rico Institutional Animal Care and Utilization Committee (Protocol #: ATP001-07-12-2013). After gentle defolliculation, oocytes were microinjected with 50 nl of 6 mg/ml of the crude membrane or 3 mg/ml of 1.5 fold critical micellar concentration of affinity purified nAChR detergent complexes from *Tc*^21^,^45^,^46^. Then, microinjected oocytes were incubated in an RO-2 solution containing sodium pyruvate supplemented with gentamicin (50 mg/ml), tetracycline (50 mg/ml), and theophylline (0.5 mM) at 18 °C for 16–36 hours. The ion channel function of *Tc* crude membrane extracts and nAChR-DCs, for each detergent above, were assayed using the TEVC approach. A 200 μl chamber containing the clamped oocyte were used, the macroscopic currents were induced by 5 seconds B7DB application of 100 μM acetylcholine (ACh), and recorded using TEVC through the GeneClamp 500B amplifier (Axon Instruments, Foster City, CA, USA). The electrodes were filled with a solution of 3 M KCl, and the resistances were calculated to average 1.3 mΩ. Acetylcholine solutions were made from calcium-depleted RO-2 to elude the activation of an endogenous Ca^2+^ dependent chloride current. Macroscopic currents were filtered at 100 Hz and digitized at 1000 Hz using a Digidata 1440A interface (Axon Instruments, Foster City, CA, USA) and acquired using the Clampex 10.2, (pCLAMP 10.2 software, Molecular Devices) running on a Microsoft Windows-based computer.

### Statistical analysis

All data was processed, and statistical analyses were conducted using the GraphPad Prism 6 software (GraphPad Software, San Diego, CA, www.graphpad.com). All samples were analyzed separately using Two-way mixed model ANOVA and One-way ANOVA. Comparison of the means for the individual treatments was made at the 5% significance level based on the *F*-test of the analysis of variance.

## Additional Information

**How to cite this article**: Quesada, O. *et al*. Uncovering the lipidic basis for the preparation of functional nicotinic acetylcholine receptor detergent complexes for structural studies. *Sci. Rep.* **6**, 32766; doi: 10.1038/srep32766 (2016).

## Supplementary Material

Supplementary Information

## Figures and Tables

**Table 1:** Total percent of fatty acids from acid and base catalyzed hydrolysis for both B&D and Foch lipid extraction methods.

Fatty Acid	MW Exact mass	Acid-Catalyzed Hydrolysis		Base-Catalyzed Hydrolysis	
		Bligh & Dyer	Folch	Bligh & Dyer	Folch
14:0	242.2246	2.0 ± 0.4	1.7 ± 0.2	2.0 ± 0.1	1.7 ± 0.1
16:0	270.2558	25.1 ± 2.7	25.0 ± 1.9	26.1 ± 0.9	20.2 ± 0.4
16:1	268.2402	5.8 ± 1.0	5.8 ± 0.5	7.5 ± 0.4	7.9 ± 0.1
18:0	298.2871	14.7 ± 3.2	16.6 ± 2.4	4.4 ± 0.1	3.1 ± 0.1
18:1	296.2715	16.8 ± 2.1	17.4 ± 0.6	17.5 ± 0.2	21.0 ± 0.5
18:2	294.2559	1.0 ± 0.2	1.2 ± 0.4	1.2 ± 0.2	2.1 ± 0.1
20:3	320.2715	0.4 ± 0.1	0.5 ± 0.1	0.5 ± 0.1	0.6 ± 0.1
20:4	318.2559	5.0 ± 0.1	4.7 ± 0.3	6.0 ± 0.1	7.8 ± 0.4
20:5	316.2402	2.7 ± 0.1	2.4 ± 0.2	2.8 ± 0.1	3.3 ± 0.1
22:1	352.2421	1.0 ± 0.1	0.9 ± 0.1	1.0 ± 0.1	0.8 ± 0.1
22:5	344.2715	2.3 ± 0.1	2.2 ± 0.1	2.7 ± 0.1	2.8 ± 0.1
22:6	342.2559	21.0 ± 0.6	19.9 ± 2.3	25.6 ± 0.6	25.5 ± 0.2
24:1	380.2540	2.2 ± 0.2	1.8 ± 0.1	2.7 ± 0.1	3.2 ± 0.1

Data are the means and ± SEM of three (3) independent experiments performed in triplicate on separate days and compared using an unpaired t-test in Graph Pad Prism 6. The information in this table is a combined data from GC-MS and GCFID of *Tc* electric organ crude membranes.

**Table 2:** Cross-correlation of major lipid species present in six nAChR-DCs from *Tc* electric organ solubilized with FC and LFC detergent family.

m/z (M+H)+	Molecular Species	DC Displaying Significant Functionality	DC’s Displaying High Functionality		DC’s Displaying Low Functionality			R*
		FC12	LFC14	LFC16	LFC12	FC 14	FC 16	
760.5825	PC 16:0/18:1	X	X	X	X	X	X	>3%
806.5697	PC 16:0/22:6	X	X	X	X	X	X	>3%
718.5746	PC(O-16:0:/16:1)	X	X	X	X	X	X	>3%
746.6097	PC(O-16:1/18:0)	X	X	X	X	X	X	~1%
746.6097	PC(O-18:0/16:1)	X	X	X	X	X	X	~1%
813.6835	SM d18:1/24:1	X	X	X	X	X	X	>3%
704.5217	PC 14:0/16:1	X						>3%
730.5325	PC 14:0/18:2			X				>3%
808.5896	PC 16:0/22:5			X				>3%
810.5925	PC 18:0/20:4		X	X	X			<1%
828.5564	PC 20:4/20:5		X	X	X			<1%
828.5564	PC 20:5/20:4		X	X	X			<1%
785.6594	SM d18:1/22:1						X	<1%
496.3445	LPC 16:0		X	X	X			<1%
468.3097	LPC 14:0		X					<1%
440.2803	LPC 12:0				X			<1%

The phospholipid composition of affinity purified nAChR detergent complexes was separated using HILIC column and analyzed by ESI-MS/MS.

R* Relative abundance in MS of crude membrane preparation. <1% indicates components found at trace levels, ~1% minor components and >3% major ones.

**Table 3:** Acetylcholine-evoked responses in oocytes injected with detergent-solubilized affinity purified nAChR detergent complexes.

	Mean Current Amplitude (nA)	Normalized Current	Rise Time 10–90% (s)	Decay Time 90–10% (s)	Number (n)
Crude	−275 ± 24	1.00 ± 0.09	1.2 ± 0.22	6.3 ± 0.50	25
FC-12	−217 ± 22	0.79 ± 0.08	3.81 ± 0.30***	6.0 ± 1.00	16
FC-14	−77 ± 18***	0.28 ± 0.07***	3.92 ± 0.38	3.3 ± 0.13***	7
FC-16	−28 ± 7***	0.08 ± 0.03***	ND	ND	17
LFC-12	−25 ± 8***	0.10 ± 0.02***	ND	ND	9
LFC-14	−420 ± 45**	1.53 ± 0.16**	3.29 ± 0.42	4.5 ± 1.50	9
LFC-16	−344 ± 25	1.25 ± 0.09	2.54 ± 0.74	6.5 ± 3.50	9

Responses were evoked by a 5-second application of 100 μM ACh. The following detergents were used FC-12, FC-14, FC-16, LFC-12, LFC-14, and LFC-16. The amplitude of the responses was compared based on their mean amplitude and then normalized to the response of the respective crude membranes used for solubilization. The kinetics of activation and deactivation of the responses were calculated using a 10–90% rise time and a 90-10% decay time and compared. Data is presented as mean ± SEM and compared using an unpaired t-test; ***P ≤ 0.0001; **P ≤ 0.001.
