# Supplementary material for: Uncovering the lipidic basis for the preparation of functional nicotinic acetylcholine receptor detergent complexes for structural studies
Source: Sci Rep. 2016 Sep 19;6:32766. doi: 10.1038/srep32766 (PMC5027579; doi:10.1038/srep32766)
Supplement: Supplementary Information [file srep32766-s1.doc]

**Uncovering the lipidic basis for the preparation of functional nicotinic acetylcholine receptor detergent complexes for structural studies**

Orestes Quesada, Carol González-Freire, María Carla Ferrer, José O. Colón-Sáez, Emily Fernández-García, Juan Mercado, Alejandro Dávila, Reginald Morales and José A. Lasalde-Dominicci

**Supplementary Fig. 1** **Thin layer chromatography of total phospholipids extracted from *Tc* electric organ using Bligh and Dyer method.** Lipids were separated by one-dimensional TLC using chloroform/methanol/ammonium hydroxide (65/25/4, v/v/v) as described in Materials and Methods section. Phospholipids standard such as phosphatidic acid (PA), phosphatidylcholine (PC), phosphatidylethanolamine (PE), phosphatidylglycerol (PG) phosphatidylserine (PS) phosphatidylinositol (PI), sphingomyelin (SM), fatty acids and Cholesterol were used for identification of individual *Tc* lipids iodine vapors stained spot.

**Supplementary Fig. 2 Hydrophilic liquid interaction chromatography**-**positive electrospray ionization mass spectrometry chromatograms of lipid components in the Bligh and Dyer extracted from *Tc* electric organ.** HILIC column optimized following the described conditions in Material and Methods section was able to separate phospholipids based on their polar head group. Eleven peaks were identified within runtime of 14 min. tri and diacylglycerol, cholesterol esters and PG, PI, PE, LPE, PC, SM, and LPC. Phosphatidylcholine head group containing phosphoglycerides (PC, SM, and LPC) eluted after 9.3 min, 10.8 min, and 11.9 min, respectively.

**Supplementary Fig. 3** **UPLC-ESI-MS-MS spectra of phospholipids found in *Tc* electric organ.** (**a**) 18:0/18:2 phosphatidylcholine quasi-molecular ion [M+H]+ at m/z 786.6077; (**b**) 18:1/20:4 alkenyl/acyl phosphatidylethanolamine quasi-molecular ions [M+H]+ at m/z 750.5478, 18:0/22:6; (**c**) phosphatidylethanolamine quasi-molecular ion [M+H]+ at m/z 7925.5566; (**d**) 16:0/18:2 phosphatidylinositol quasi-molecular ion [M-H]- at m/z 833.5163; (**e**) 16:0/18:2 phosphatidylgycerol [M-H]- at m/z 845.4993 and (**f**) 16:0/18:2 phosphatidylserine [M-H]- at m/z 758.4943.

**Supplementary Fig. 4 Original SDS-PAGE of nAChR detergent complexes with two lipid-like detergent families containing 12-16 carbon chain length.** Affinity purified nAChR detergent complex for the six (6) lipid-like detergents and the nAChR-enriched crude membrane from *Torpedo californica* at different dilution ratio were analyzed in the same gel under identical conditions and concentrations.

**Supplementary Table 1** **Molecular species of phospholipid found in the extracted lipids from *Tc*electric organ using Bligh & Dyer extraction method.**The molecular composition of all detected phospholipid classes from of *Tc* electric organ extracted using the B&D method were analyzed by UPLC ESI Q-Tof MS/MS in positive and negative resolution mode.

**Supplementary Fig. 1**


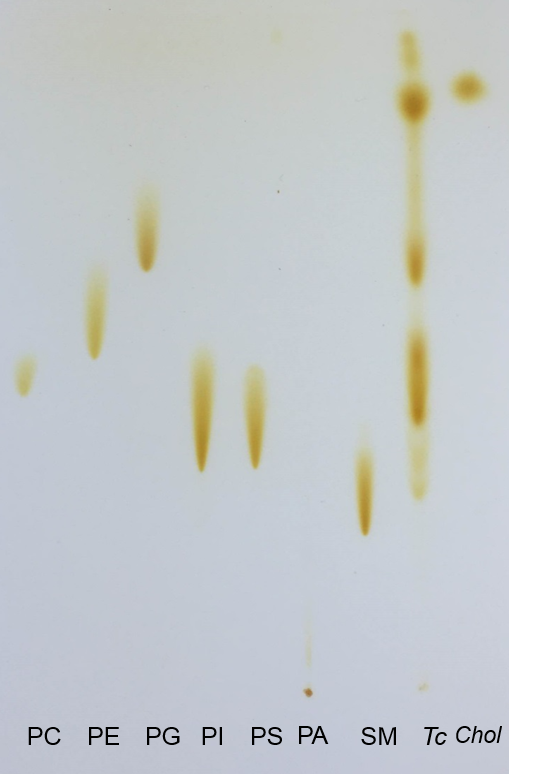


**Supplementary Fig. 2**


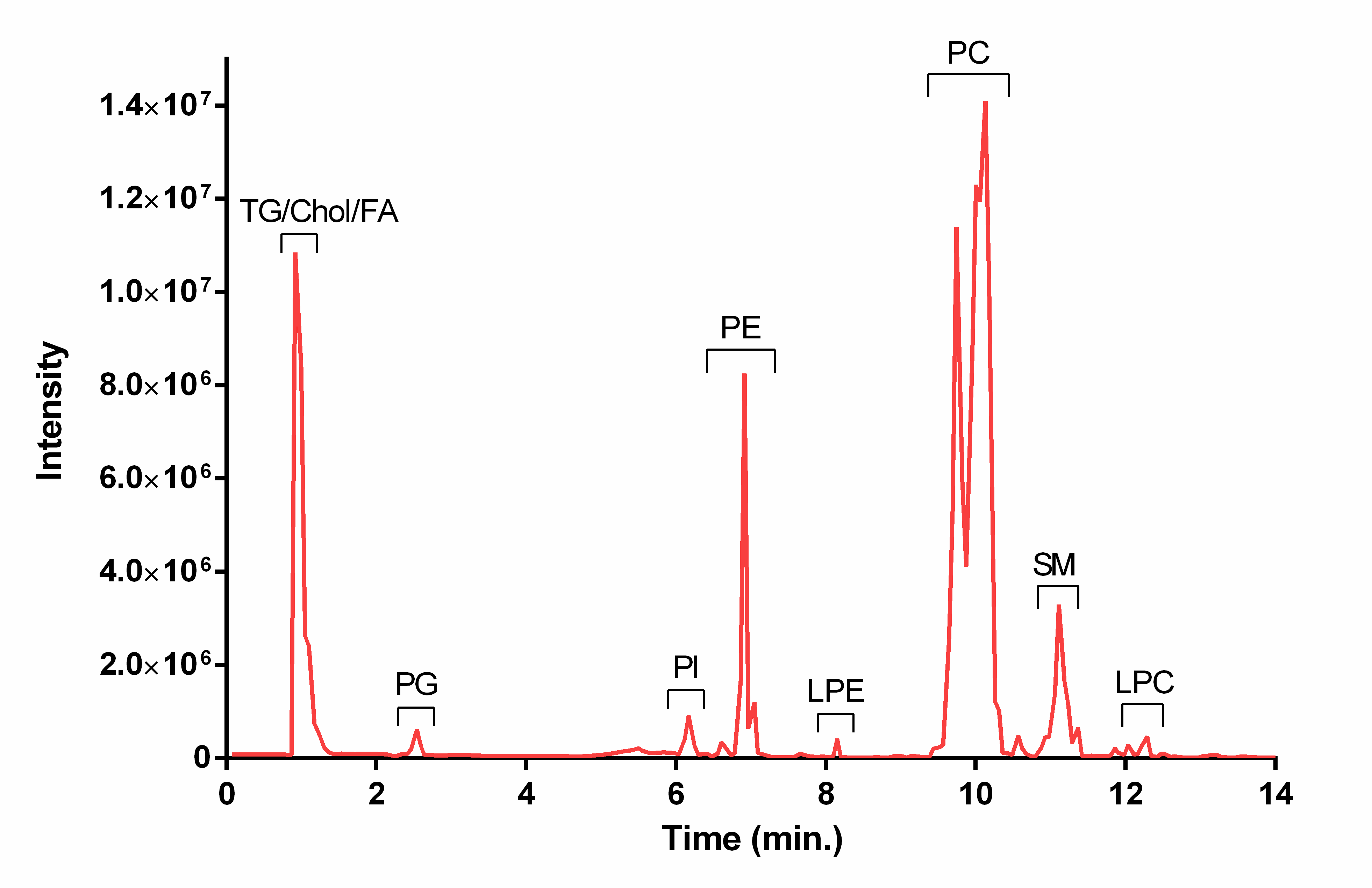


**Supplementary Fig. 3**


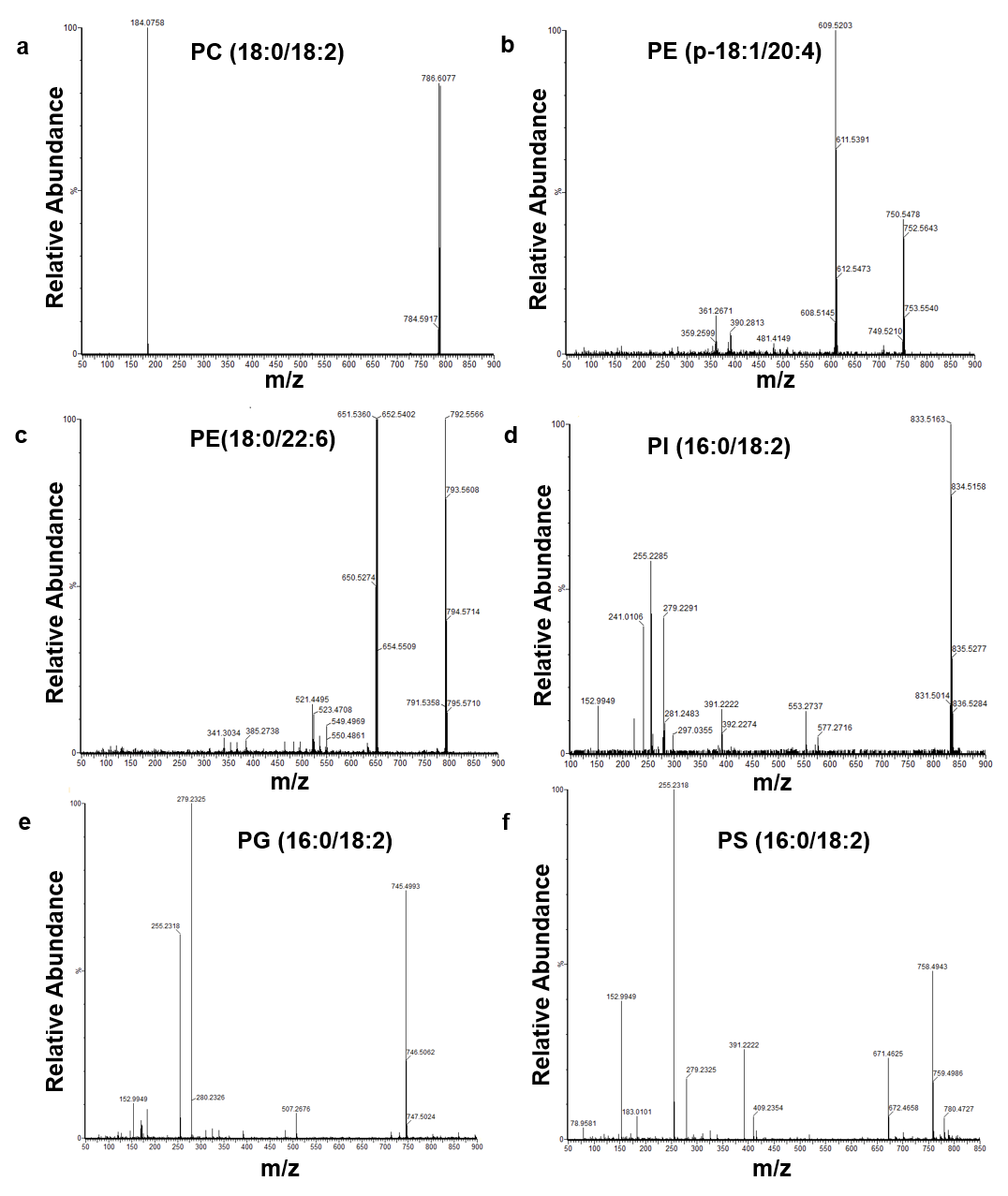


**Supplementary Fig. 4**

**
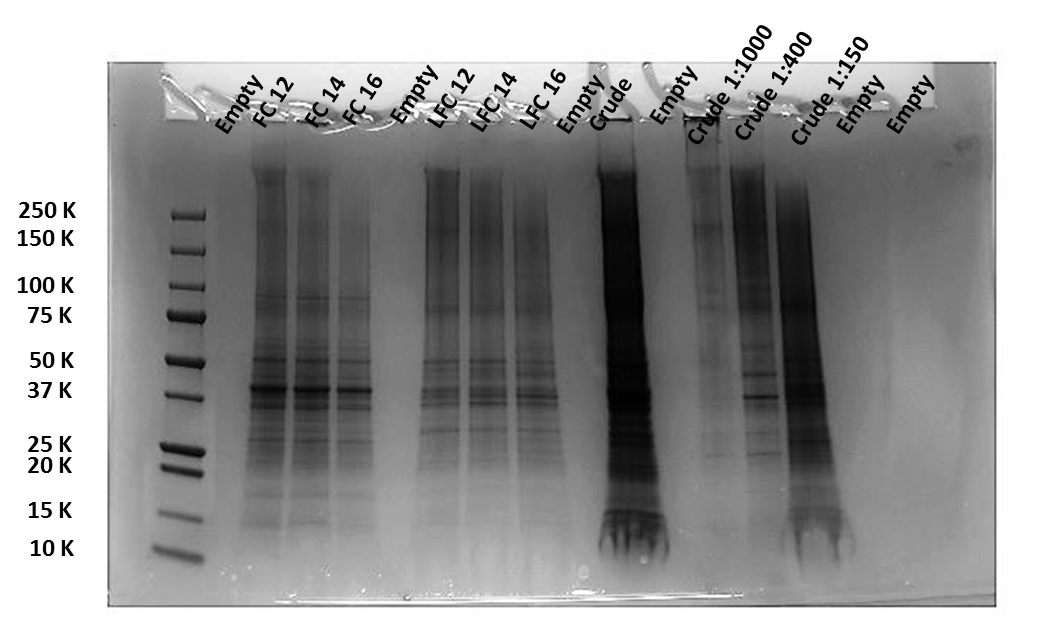
**

**Supplementary Table 1.**

| **m/z** | **Relative intensity** | **Specie** | **Exact mass** | **Formula** | **Error (ppm)** | **MS/MS Product Ions** |
| --- | --- | --- | --- | --- | --- | --- |
| **PC [M+H]+** |  |  |  |  |  |  |
| 704.5184 | 20.44 | PC 14:0/16:1 | 704.5225 | C_38_H_75_NO_8_P | 5.8 | 704,184 |
| 704.5216 | 0.48 | PC 16:0/14:1 | 704.5225 | C_38_H_75_NO_8_P | 1.3 | 704,184 |
| 718.5746 | 40.67 | PC(O-16:0:/16:1) | 718.5745 | C_40_H_81_NO_7_P | 0.1 | 718,480,184 |
| 728.5290 | 2.60 | PC 18:2/14.1 | 728.5236 | C_40_H_75_NO_8_P | 7.4 | 728,504,184 |
| 730.5325 | 20.54 | PC 14:0/18:2 | 730.5381 | C_40_H_77_NO_8_P | 7.7 | 730,468,184 |
| 732.5514 | 20.54 | PC 14:0/18:1 | 732.5538 | C_40_H_79_NO_8_P | 3.3 | 732,468,184 |
| 732.5548 | 1.08 | PC 16:0/16:1 | 732.5538 | C_48_H_83_NO_8_P | 1.4 | 732,496,184 |
| 746.6097 | 3.13 | PC(O-16:1/18:0) | 746.6058 | C_42_H_85_NO_7_P | 5.2 | 746,184 |
| 746.6097 | 3.13 | PC(O-18:0/16:1) | 746.6058 | C_42_H_85_NO_7_P | 5.2 | 746,184 |
| 750.5343 | 2.25 | PC14:1/20:5 | 750.5068 | C_42_H_73_NO_8_P | 3.3 | 750,466,184 |
| 758.5604 | 7.12 | PC 16:0/18:2 | 758.5694 | C_42_H_81_NO_8_P | 11.9 | 758,496,184 |
| 760.5825 | 100.00 | PC 16:0/18:1 | 760.5851 | C_42_H_83_NO_8_P | 3.4 | 760,496,184 |
| 776.5196 | 1.02 | PC 14:1/22:6 | 776.5225 | C_44_H_75_NO_8_P | 3.7 | 776,466,184 |
| 778.5379 | 0.95 | PC 14:0/22:6 | 778.5381 | C_44_H_77_NO_8_P | 0.3 | 760,468,184 |
| 782.5698 | 9.38 | PC 16:0/20:4 | 782.5694 | C_44_H_81_NO_8_P | 0.5 | 782,496,184 |
| 786.6077 | 3.32 | PC 18:0/18:2 | 786.6007 | C_44_H_85_NO_8_P | 8.9 | 786,524,184 |
| 792.5967 | 4.15 | PC (O-16:0/22:6) | 792.5902 | C_46_H_83_NO_7_P | 8.2 | 792,551,184 |
| 806.5602 | 15.83 | PC 18:2/20:4 | 806.5694 | C_46_H_81_NO_8_P | 11.4 | 798,520,184 |
| 806.5697 | 28.11 | PC 16:0/22:6 | 806.5694 | C_46_H_84_N_2_O_8_P | 0.4 | 806,496,184 |
| 808.5896 | 12.06 | PC 16:0/22:5 | 808.5851 | C_46_H_83_NO_8_P | 5.6 | 808,496,184 |
| 810.5925 | 2.14 | PC 18:0/20:4 | 810.6007 | C_46_H_85_NO_8_P | 10.1 | 810,544,184 |
| 812.6067 | 1.83 | PC 18:0/20:3 | 812.6164 | C_46_H_87_NO_8_P | 11.9 | 814,544,184 |
| 820.6219 | 1.03 | PC (O-18:0/22:6) | 820.6215 | C_48_H_87_NO_7_P | 0.5 | 820,508,184 |
| 828.5564 | 1.23 | PC 20:4/20:5 | 828.5538 | C_48_H_79_NO_8_P | 3.1 | 828,184 |
| 828.5564 | 1.23 | PC 20:5/20:4 | 828.5538 | C_48_H_79_NO_8_P | 3.1 | 828,184 |
| 834.6044 | 2.11 | PC 18:0/22:6 | 834.6007 | C_48_H_85_NO_8_P | 4.4 | 834,524,184 |
| 836.6225 | 2.19 | PC 18:0/22:5 | 836.6164 | C_48_H_87_NO_8_P | 7.3 | 836,524,184 |
| 844.6640 | 0.59 | PC 18:0/22:1 | 844.6790 | C_48_H_95_NO_8_P | 17.8 | 844,524,184 |
| 844.6680 | 2.06 | PC 16:0/24:1 | 844.6741 | C_48_H_95_NO_8_P | 7.2 | 844,496,184 |
| 862.6478 | 0.94 | PC 22:1/20:5 | 862.6320 | C_48_H_95_NO_8_P | 18.3 | 862,578,184 |
| 872.7093 | 0.77 | PC 18:0/24:1 | 872.7103 | C_50_H_99_NO_8_P | 1.1 | 872,524,184 |
| 878.5639 | 0.02 | PC 22:6/22:6 | 878.5694 | C_52_H_81_NO_8_P | 6.3 | 878,568,184 |
| **LPC (M+H)+** |  |  |  |  |  |  |
| 496.3445 | 0.94 | LPC 16:0 | 496.3398 | C_24_H_51_NO_7_P | 9.5 | 496,184 |
| 524.3641 | 0.22 | LPC 18:0 | 524.3711 | C_26_H_55_NO_7_P | 13.3 | 524,184 |
| **SM (M+H)+** |  |  |  |  |  |  |
| 675.5479 | 24.55 | SM d18:1/14:0 | 675.5435 | C_37_H_76_N_2_O_6_P | 6.5 | 675,657,184 |
| 703.5784 | 55.28 | SM d18:1/16:0 | 703.5748 | C_39_H_80_N_2_O_6_P | 5.1 | 703,685,184 |
| 813.6835 | 27.57 | SM d18:1/24:1 | 813.6844 | C_47_H_94_N_2_O_6_P | 1.1 | 813,184 |
| 731.6048 | 2.76 | SM d18:0/16:0 | 731.6061 | C_41_H_84_N_2_O_6_P | 1.8 | 731,496,184 |
| 785.6594 | 10.04 | SM d18:1/22:1 | 785.6531 | C_45_H_90_N_2_O_6_P | 8.0 | 785,767,184 |
| **PE (M+H)+** |  |  |  |  |  |  |
| 702.5465 | 6.64 | PE (P-16:0/18:1) | 702.5432 | C_39_H_77_NO_7_P | 4.7 | 702,565,364 |
| 716.5201 | 1.40 | PE 16:0/18:2 | 716.5225 | C_39_H_75_NO_8_P | 3.3 | 716,575,454 |
| 746.5587 | 1.10 | PE 18:0/18:1 | 746.5694 | C_41_H_81_NO_8_P | 14.3 | 746,605,482 |
| 750.5478 | 14.07 | PE (P-18:1/20:4) | 750.5589 | C_45_H_81_NO_7_P | 14.8 | 750,609,390 |
| 750.5478 | 5.04 | PE (O-16:0/22:6) | 750.5432 | C_43_H_77_NO_7_P | 6.1 | 750,609,440 |
| 764.5287 | 17.08 | PE 16:0/22:6 | 764.5225 | C_43_H_75_NO_8_P | 8.1 | 764,623,526 |
| 766.5471 | 0.67 | PE 18:1/20:5 | 766.5381 | C_43_H_77_NO_8_P | 11.7 | 766,624,480 |
| 770.5623 | 1.18 | PE 18:2/22:1 | 770.5694 | C_43_H_81_NO_8_P | 9.2 | 770,629,478 |
| 774.6017 | 1.55 | PE 16:0/22:1 | 774.6007 | C_43_H_85_NO_8_P | 1.3 | 774,633,454 |
| 776.5592 | 1.04 | PE (P-18:0/22:6) | 776.5589 | C_45_H_79_NO_7_P | 0.4 | 776,635,392 |
| 786.5106 | 0.67 | PE 20:4/20:5 | 786.5068 | C_45_H_73_NO_8_P | 4.8 | 786,645,502 |
| 792.5566 | 1.04 | PE 18:0/22:6 | 792.5538 | C_45_H_79_NO_8_P | 3.5 | 792,521,385 |
| 814.5278 | 0.60 | PE 20:3/22:6 | 814.5381 | C_47_H_77_NO_8_P | 12.6 | 814,673,504 |
| 820.5869 | 1.31 | PE 20:5/22:1 | 820.5851 | C_47_H_83_NO_8_P | 2.2 | 820,679,500 |
| 822.5901 | 1.04 | PE 22:1/22:6 | 822.6007 | C_47_H_85_NO_8_P | 12.9 | 822,680,536 |
| **LPE (M+H)+** |  |  |  |  |  |  |
| 504.3025 | 0.16 | LPE 20:3 | 504.3085 | C_25_H_47_NO_7_P | 9.9 | 504,307 |
| 526.2917 | 0.05 | LPE 22:6 | 526.2928 | C_27_H_45_NO_7_P | 2.1 | 526,328 |
| 454.2954 | 0.08 | LPE 16:0 | 454.2928 | C_21_H_45_NO_7_P | 5.7 | 454,313 |
| 480.3083 | 1.01 | LPE 18:1 | 480.3085 | C_23_H_47_NO_7_P | 0.4 | 480,339 |
| **PG (M-H)-** |  |  |  |  |  |  |
| 749.5299 | 2.56 | PG 16:0/18:0 | 749.5338 | C_40_H_78_O_10_P | 5.2 | 749,255,283,171 |
| 747.5137 | 12.30 | PG 16:0/18:1 | 747.5182 | C_40_H_76_O_10_P | 6.0 | 747,255,281,171 |
| 745.4993 | 13.39 | PG 16:0/18:2 | 745.5025 | C_40_H_74_O_10_P | 4.3 | 745,255,279,171 |
| 765.4677 | 26.57 | PG 16:1/20:5 | 765.4712 | C_42_H_70_O_10_P | 4.6 | 765,253,303,171 |
| 771.5136 | 73.70 | PG 16:0/20:3 | 771.5182 | C_42_H_76_O_10_P | 6.0 | 771,255,305,171 |
| 791.4810 | 3.56 | PG 18:2/20:5 | 791.4869 | C_44_H_72_O_10_P | 7.5 | 791,279,301,171 |
| 793.4945 | 100.00 | PG 16:0/22:6 | 793.5025 | C_44_H_74_O_10_P | 10.1 | 793,255,327,171 |
| 797.5289 | 11.55 | PG 18:0/20:4 | 797.5338 | C_44_H_78_O_10_P | 6.1 | 797,283,303,171 |
| **PS (M-H)-** |  |  |  |  |  |  |
| 758.4943 | 0.46 | PS 16:0/18:2 | 758.4978 | C_40_H_73_NO_10_P | 4.6 | 758,671,255,279 |
| 786.5247 | 0.06 | PS 18:0/18:2 | 786.5291 | C_42_H_77_NO_10_P | 5.6 | 786,699,283,279 |
| 834.5199 | 0.51 | PS 18:0/22:6 | 834.5291 | C_46_H_77_NO_10_P | 11.0 | 834,747,283,327 |
| 856.5087 | 0.16 | PS 20:3/22:6 | 856.5134 | C_48_H_75_NO_10_P | 5.5 | 856,769,305,327 |
| **PI (M-H)-** |  |  |  |  |  |  |
| 833.5163 | 0.44 | PI 16:0/18:2 | 833.5186 | C_43_H_78_O_13_P | 2.8 | 833,253,279,241 |
| 835.5299 | 1.10 | PI 16:0/18:1 | 835.5342 | C_43_H_80_O_13_P | 5.1 | 835,255,281,241 |
| 859.5311 | 0.67 | PI 16:0/20:3 | 859.5342 | C_45_H_80_O_13_P | 3.6 | 859,255,305 ,241 |
| 881.5095 | 1.16 | PI 16:0/22:6 | 881.5186 | C_47_H_78_O_13_P | 10.3 | 881,255,327,241 |
| 885.5464 | 0.28 | PI 18:1/20:3 | 885.5499 | C_47_H_82_O_13_P | 4.0 | 885,281,305,241 |
| 909.5417 | 0.13 | PI 18:0/22:6 | 909.5499 | C_49_H_82_O_13_P | 9.0 | 909,283,327,241 |
| 931.5300 | 1.95 | PI 20:3/22:6 | 931.5342 | C_51_H_80_O_13_P | 4.5 | 931,305,327,241 |
